# Bis(ethano­laminium) succinate–succinic acid (1/1)

**DOI:** 10.1107/S1600536811034428

**Published:** 2011-08-27

**Authors:** Miao Zhang, Cong Wang, Zheng Fan

**Affiliations:** a College of Pharmaceutical Sciences, Zhejiang University of Technology, Hangzhou 310014, People’s Republic of China; b College of Biological and Environmental Engineering, Zhejiang University of Technology, Hangzhou 310014, People’s Republic of China

## Abstract

The asymmetric unit of the title compound, 2C_2_H_8_NO^+^·C_4_H_4_O_4_^2−^·C_4_H_6_O_4_, consists of half a succinate anion, half a succinic acid mol­ecule and one ethano­laminium cation. The succinate anion and succinic acid mol­ecule, both of which are located on inversion centres, are linked by O—H⋯O hydrogen bonds, forming a chain along the [2
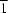
0] direction. The chain and the ethano­laminium cation are further connected by O—H⋯O and N—H⋯O hydrogen bonds.

## Related literature

For related structures of co-crystals and salts of succinic acid, see: Aakeroy *et al.* (1998 ▶); Batchelor *et al.* (2001 ▶); Borthwick (1980 ▶); Braga *et al.* (2003 ▶); Bruno *et al.* (2004 ▶); Büyükgüngör & Odabasoglu (2002 ▶); Flensburg *et al.* (1995 ▶); Kuipers *et al.* (1997 ▶); Li *et al.* (2003 ▶); MacDonald *et al.* (2001 ▶); Prasad & Vijayan (1990 ▶); Reitz *et al.* (1998 ▶); Urbanczyk-Lipkowska (2000 ▶).
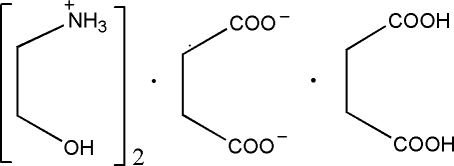


## Experimental

### 

#### Crystal data

2C_2_H_8_NO^+^·C_4_H_4_O_4_^2−^·C_4_H_6_O_4_
*M* *_r_* = 358.35
Triclinic, 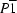

*a* = 5.821 (5) Å
*b* = 8.428 (7) Å
*c* = 9.077 (7) Å
α = 87.74 (1)°
β = 73.628 (11)°
γ = 80.380 (12)°
*V* = 421.2 (6) Å^3^
*Z* = 1
Mo *K*α radiation
μ = 0.12 mm^−1^
*T* = 293 K
0.42 × 0.34 × 0.30 mm

#### Data collection

Bruker APEX area-detector diffractometer
Absorption correction: multi-scan (*SADABS*; Bruker, 2001 ▶) *T*_min_ = 0.855, *T*_max_ = 0.898
2330 measured reflections
1628 independent reflections
1517 reflections with *I* > 2σ(*I*)
*R*_int_ = 0.011

#### Refinement

*R*[*F* ^2^ > 2σ(*F* ^2^)] = 0.036
*wR*(*F* ^2^) = 0.106
*S* = 1.07
1628 reflections
110 parameters
H-atom parameters constrained
Δρ_max_ = 0.37 e Å^−3^
Δρ_min_ = −0.32 e Å^−3^

### 

Data collection: *SMART* (Bruker, 2007 ▶); cell refinement: *SAINT* (Bruker, 2007 ▶); data reduction: *SAINT*; program(s) used to solve structure: *SHELXS97* (Sheldrick, 2008 ▶); program(s) used to refine structure: *SHELXL97* (Sheldrick, 2008 ▶); molecular graphics: *SHELXTL* (Sheldrick, 2008 ▶); software used to prepare material for publication: *SHELXL97*.

## Supplementary Material

Crystal structure: contains datablock(s) global, I. DOI: 10.1107/S1600536811034428/is2756sup1.cif

Structure factors: contains datablock(s) I. DOI: 10.1107/S1600536811034428/is2756Isup2.hkl

Supplementary material file. DOI: 10.1107/S1600536811034428/is2756Isup3.cml

Additional supplementary materials:  crystallographic information; 3D view; checkCIF report

## Figures and Tables

**Table 1:** Hydrogen-bond geometry (Å, °)

*D*—H⋯*A*	*D*—H	H⋯*A*	*D*⋯*A*	*D*—H⋯*A*
N1—H1*A*⋯O1^i^	0.89	2.09	2.976 (2)	171
N1—H1*B*⋯O3^ii^	0.89	1.93	2.810 (2)	167
N1—H1*C*⋯O5	0.89	2.55	2.868 (3)	102
N1—H1*C*⋯O5^iii^	0.89	2.31	2.913 (3)	125
O2—H2⋯O4	0.82	1.66	2.466 (2)	166
O5—H5⋯O4	0.82	2.01	2.697 (2)	142

Symmetry codes: (i) 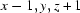; (ii) 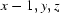; (iii) 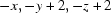.

**Fig. 1.**
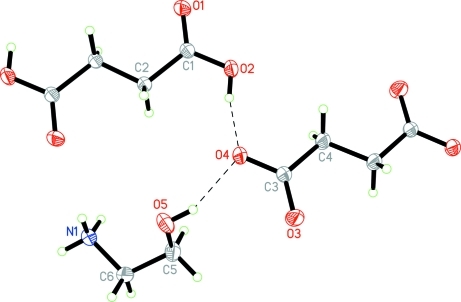
The molecular structure of the title compound. Hydrogen bonds are illustrated as dashed lines.

**Fig. 2.**
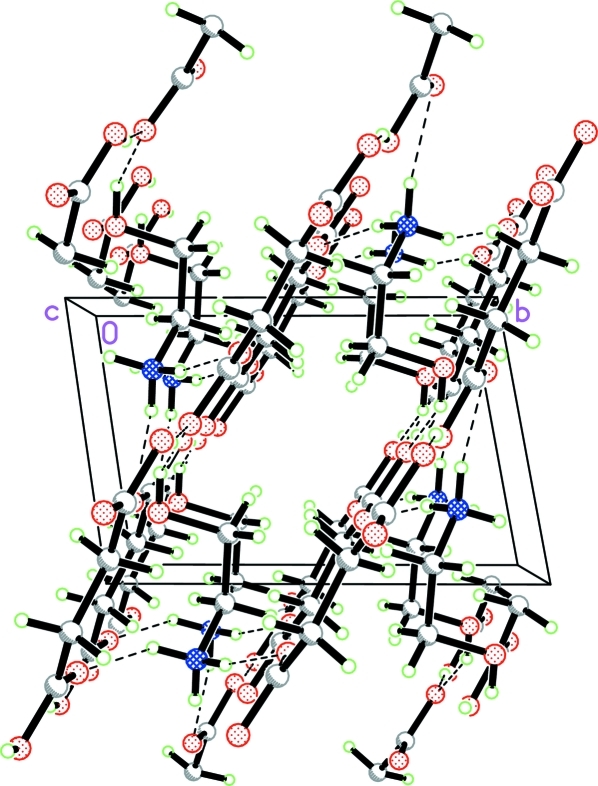
The crystal packing of the title compound, viewed down the c axis. Hydrogen bonds are drawn as dashed lines.

## supplementary crystallographic information

### Comment

There exist three forms of succinic acid in molecular complex, namely, succinate (Kuipers *et al.*, 1997; Urbanczyk-Lipkowska, 2000), succinic acid (Aakeroy *et al.*, 1998; Batchelor *et al.*, 2001), hydrogen succinate (Flensburg *et al.*, 1995; MacDonald *et al.*, 2001). Interestingly, some mixed forms are also available (Prasad & Vijayan, 1990; Reitz *et al.*, 1998; Büyükgüngör & Odabasoglu, 2002; Braga *et al.*, 2003; Bruno *et al.*, 2004). However, no convincing explanation for the formation of any of the complex. Recently, the title complex, (I) (Table 1 & Fig. 1), is synthesized, and the structure is studied hereafter.

As shown in Fig. 1, the asymmetric unit is composed of one ethanolaminium cation, half a succinate anion, and half a succinic acid molecule. The succinate anion and succinic acid molecule are linked by an O2—H2···O4 hydrogen bond (Table 1), then are associated with ethanolaminium cation by an O5—H5···O4 hydrogen bond. The distances of [C1—O1 1.2187 (18) Å and C1—O2 1.3009 (18) Å] indicate a carboxylic group, where C1—O1 stands for the carbonyl C=O bond. The distances of [C3—O3 1.2310 (18) Å and C3—O4 1.2831 (17) Å] indicate a carboxylate anion, where the C—O and C=O bonds are equalized, and no distinct carbonyl C=O bond is observed (Borthwick, 1980). This is mixed mode of succinate and succinic acid, and is similar to those observed in other cases (Prasad & Vijayan, 1990; Li *et al.*, 2003; Bruno *et al.*, 2004).

The succinate anion and the succinic acid molecule are arranged almost in the same layers (Fig. 2), and the ethanolaminium cation are sandwiched between the layers by O—H···O and N—H···O hydrogen bonds (Table 1).

### Experimental

Succinic acid (5.1 g) and ethanolamine (2.4 g), in a molar ratio of 1:1, were mixed and dissolved in sufficient ethanol by heating to 373 K, at which point a clear solution resulted. The system was then cooled slowly to room temperature. Crystals of (I) (4.3 g) were formed, collected and washed with ethanol.

### Refinement

All H atoms were placed in calculated positions and allowed to ride on their parent atoms with distances of 0.89 Å for the amido, 0.97 Å for the methylene and 0.82 Å for the hydroxyl group, and with isotropic displacement parameters 1.2–1.5 times *U*_eq_ of the parent atoms.

### Crystal data

**Table:**

2C_2_H_8_NO^+^·C_4_H_4_O_4_^2^^−^·C_4_H_6_O_4_	*Z* = 1
*M**_r_* = 358.35	*F*(000) = 192.0
Triclinic, *P*1	*D*_x_ = 1.413 Mg m^−^^3^
Hall symbol: -P 1	Mo *K*α radiation, λ = 0.71073 Å
*a* = 5.821 (5) Å	Cell parameters from 1360 reflections
*b* = 8.428 (7) Å	θ = 2.1–17.8°
*c* = 9.077 (7) Å	µ = 0.12 mm^−^^1^
α = 87.74 (1)°	*T* = 293 K
β = 73.628 (11)°	Prism, colorless
γ = 80.380 (12)°	0.42 × 0.34 × 0.30 mm
*V* = 421.2 (6) Å^3^	

### Data collection

**Table:**

Bruker APEX area-detector diffractometer	1628 independent reflections
Radiation source: fine-focus sealed tube	1517 reflections with *I* > 2σ(*I*)
graphite	*R*_int_ = 0.011
φ and ω scan	θ_max_ = 26.0°, θ_min_ = 2.3°
Absorption correction: multi-scan (*SADABS*; Bruker, 2001)	*h* = −4→7
*T*_min_ = 0.855, *T*_max_ = 0.898	*k* = −9→10
2330 measured reflections	*l* = −11→10

### Refinement

**Table:**

Refinement on *F*^2^	Secondary atom site location: difference Fourier map
Least-squares matrix: full	Hydrogen site location: inferred from neighbouring sites
*R*[*F*^2^ > 2σ(*F*^2^)] = 0.036	H-atom parameters constrained
*wR*(*F*^2^) = 0.106	*w* = 1/[σ^2^(*F*_o_^2^) + (0.0582*P*)^2^ + 0.1261*P*] where *P* = (*F*_o_^2^ + 2*F*_c_^2^)/3
*S* = 1.07	(Δ/σ)_max_ < 0.001
1628 reflections	Δρ_max_ = 0.37 e Å^−^^3^
110 parameters	Δρ_min_ = −0.32 e Å^−^^3^
0 restraints	Extinction correction: *SHELXL*, Fc^*^=kFc[1+0.001xFc^2^λ^3^/sin(2θ)]^-1/4^
Primary atom site location: structure-invariant direct methods	Extinction coefficient: 0.160 (15)

### Special details

**Table:**

Geometry. All e.s.d.'s (except the e.s.d. in the dihedral angle between two l.s. planes) are estimated using the full covariance matrix. The cell e.s.d.'s are taken into account individually in the estimation of e.s.d.'s in distances, angles and torsion angles; correlations between e.s.d.'s in cell parameters are only used when they are defined by crystal symmetry. An approximate (isotropic) treatment of cell e.s.d.'s is used for estimating e.s.d.'s involving l.s. planes.
Refinement. Refinement of *F*^2^ against ALL reflections. The weighted *R*-factor *wR* and goodness of fit *S* are based on *F*^2^, conventional *R*-factors *R* are based on *F*, with *F* set to zero for negative *F*^2^. The threshold expression of *F*^2^ > σ(*F*^2^) is used only for calculating *R*-factors(gt) *etc*. and is not relevant to the choice of reflections for refinement. *R*-factors based on *F*^2^ are statistically about twice as large as those based on *F*, and *R*- factors based on ALL data will be even larger.

### Fractional atomic coordinates and isotropic or equivalent isotropic displacement parameters (Å^2^)

**Table:**

	*x*	*y*	*z*	*U*_iso_*/*U*_eq_	
O1	0.27229 (18)	0.92694 (13)	0.23761 (10)	0.0366 (3)	
O2	0.49374 (19)	0.79510 (15)	0.37464 (11)	0.0455 (3)	
H2	0.4875	0.7808	0.4655	0.068*	
O3	0.7920 (2)	0.61078 (13)	0.76185 (11)	0.0410 (3)	
O4	0.54895 (19)	0.74043 (13)	0.63248 (11)	0.0401 (3)	
O5	0.24811 (17)	0.85165 (11)	0.90544 (11)	0.0341 (3)	
H5	0.3825	0.8324	0.8434	0.051*	
N1	−0.25612 (19)	0.82179 (14)	1.00467 (12)	0.0293 (3)	
H1A	−0.4002	0.8428	1.0740	0.044*	
H1B	−0.2650	0.7570	0.9321	0.044*	
H1C	−0.2131	0.9133	0.9624	0.044*	
C1	0.2989 (2)	0.89023 (16)	0.36369 (14)	0.0263 (3)	
C2	0.1142 (2)	0.95017 (16)	0.51200 (14)	0.0275 (3)	
H2A	0.1871	1.0147	0.5668	0.033*	
H2B	0.0709	0.8586	0.5756	0.033*	
C3	0.7343 (2)	0.63837 (16)	0.64176 (14)	0.0275 (3)	
C4	0.8844 (2)	0.55193 (17)	0.49388 (15)	0.0328 (3)	
H4A	0.7865	0.4850	0.4619	0.039*	
H4B	0.9250	0.6314	0.4146	0.039*	
C5	0.1738 (2)	0.70523 (16)	0.96716 (17)	0.0336 (3)	
H5A	0.2895	0.6486	1.0177	0.040*	
H5B	0.1672	0.6370	0.8854	0.040*	
C6	−0.0726 (2)	0.74220 (17)	1.08064 (15)	0.0301 (3)	
H6A	−0.1207	0.6431	1.1278	0.036*	
H6B	−0.0653	0.8120	1.1610	0.036*	

### Atomic displacement parameters (Å^2^)

**Table:**

	*U*^11^	*U*^22^	*U*^33^	*U*^12^	*U*^13^	*U*^23^
O1	0.0321 (5)	0.0531 (7)	0.0204 (5)	0.0042 (4)	−0.0067 (4)	0.0002 (4)
O2	0.0322 (6)	0.0685 (8)	0.0230 (5)	0.0212 (5)	−0.0038 (4)	−0.0017 (5)
O3	0.0455 (6)	0.0490 (6)	0.0238 (5)	0.0142 (5)	−0.0135 (4)	−0.0085 (4)
O4	0.0341 (6)	0.0510 (6)	0.0240 (5)	0.0185 (5)	−0.0041 (4)	−0.0040 (4)
O5	0.0315 (5)	0.0336 (5)	0.0291 (5)	−0.0030 (4)	0.0042 (4)	−0.0051 (4)
N1	0.0265 (6)	0.0322 (6)	0.0259 (6)	−0.0004 (4)	−0.0040 (4)	−0.0022 (4)
C1	0.0226 (6)	0.0327 (7)	0.0218 (6)	−0.0008 (5)	−0.0052 (5)	−0.0003 (5)
C2	0.0227 (7)	0.0359 (7)	0.0204 (6)	0.0027 (5)	−0.0047 (5)	0.0002 (5)
C3	0.0268 (6)	0.0303 (7)	0.0221 (6)	0.0030 (5)	−0.0057 (5)	−0.0021 (5)
C4	0.0305 (7)	0.0401 (8)	0.0240 (7)	0.0103 (6)	−0.0096 (6)	−0.0077 (6)
C5	0.0284 (7)	0.0299 (7)	0.0387 (8)	0.0014 (5)	−0.0066 (6)	−0.0008 (6)
C6	0.0296 (7)	0.0349 (7)	0.0250 (6)	−0.0038 (5)	−0.0075 (5)	0.0030 (5)

### Geometric parameters (Å, °)

**Table:**

O1—C1	1.2187 (18)	C2—C2^i^	1.516 (2)
O2—C1	1.3009 (18)	C2—H2A	0.9700
O2—H2	0.8200	C2—H2B	0.9700
O3—C3	1.2310 (18)	C3—C4	1.5162 (19)
O4—C3	1.2831 (17)	C4—C4^ii^	1.508 (3)
O5—C5	1.4181 (19)	C4—H4A	0.9700
O5—H5	0.8200	C4—H4B	0.9700
N1—C6	1.4851 (18)	C5—C6	1.503 (2)
N1—H1A	0.8900	C5—H5A	0.9700
N1—H1B	0.8900	C5—H5B	0.9700
N1—H1C	0.8900	C6—H6A	0.9700
C1—C2	1.5097 (19)	C6—H6B	0.9700
			
C1—O2—H2	109.5	O4—C3—C4	116.06 (11)
C5—O5—H5	109.5	C4^ii^—C4—C3	114.11 (14)
C6—N1—H1A	109.5	C4^ii^—C4—H4A	108.7
C6—N1—H1B	109.5	C3—C4—H4A	108.7
H1A—N1—H1B	109.5	C4^ii^—C4—H4B	108.7
C6—N1—H1C	109.5	C3—C4—H4B	108.7
H1A—N1—H1C	109.5	H4A—C4—H4B	107.6
H1B—N1—H1C	109.5	O5—C5—C6	108.92 (11)
O1—C1—O2	119.92 (12)	O5—C5—H5A	109.9
O1—C1—C2	123.11 (12)	C6—C5—H5A	109.9
O2—C1—C2	116.97 (11)	O5—C5—H5B	109.9
C1—C2—C2^i^	113.14 (13)	C6—C5—H5B	109.9
C1—C2—H2A	109.0	H5A—C5—H5B	108.3
C2^i^—C2—H2A	109.0	N1—C6—C5	111.06 (12)
C1—C2—H2B	109.0	N1—C6—H6A	109.4
C2^i^—C2—H2B	109.0	C5—C6—H6A	109.4
H2A—C2—H2B	107.8	N1—C6—H6B	109.4
O3—C3—O4	123.24 (12)	C5—C6—H6B	109.4
O3—C3—C4	120.70 (12)	H6A—C6—H6B	108.0

Symmetry codes: (i) −*x*, −*y*+2, −*z*+1; (ii) −*x*+2, −*y*+1, −*z*+1.

### Hydrogen-bond geometry (Å, °)

**Table:**

*D*—H···*A*	*D*—H	H···*A*	*D*···*A*	*D*—H···*A*
N1—H1A···O1^iii^	0.89	2.09	2.976 (2)	171
N1—H1B···O3^iv^	0.89	1.93	2.810 (2)	167
N1—H1C···O5	0.89	2.55	2.868 (3)	102
N1—H1C···O5^v^	0.89	2.31	2.913 (3)	125
O2—H2···O4	0.82	1.66	2.466 (2)	166
O5—H5···O4	0.82	2.01	2.697 (2)	142

Symmetry codes: (iii) *x*−1, *y*, *z*+1; (iv) *x*−1, *y*, *z*; (v) −*x*, −*y*+2, −*z*+2.
